# Chinese herbal medicine for the treatment of children with cerebral palsy: a meta-analysis of randomized controlled trials with core herbs exploration

**DOI:** 10.3389/fphar.2025.1500095

**Published:** 2025-02-26

**Authors:** Ying-Yu Huang, Ya-Yun Cheng, Hsing-Yu Chen, Ren-Huei Fu, Yi-Jung Chang, Tsung-Hsien Yang

**Affiliations:** ^1^ Division of Chinese Internal and Pediatric Medicine, Center for Traditional Chinese Medicine, Chang Gung Memorial Hospital, Taipei, Taiwan; ^2^ Division of Chinese Acupuncture and Traumatology, Center of Traditional Chinese Medicine, Chang Gung Memorial Hospital, Taoyuan, Taiwan; ^3^ School of Traditional Chinese Medicine, College of Medicine, Chang Gung University, Taoyuan, Taiwan; ^4^ Department of Pediatrics and Neonatology, Chang Gung Memorial Hospital, Linkou, Taiwan; ^5^ Department of Pediatrics, Chang Gung Memorial Hospital, Chang Gung University College of Medicine, Taoyuan, Taiwan

**Keywords:** meta-analysis, cerebral palsy, Chinese herbal medicine, system pharmacology, traditional Chinese medicine

## Abstract

**Introduction:**

Chinese herbal medicine (CHM) taken orally is frequently utilized to enhance functional ability and independence in cerebral palsy (CP); nonetheless, there is a lack of current evidence regarding the efficacy of oral CHM in treating CP. Additionally, the general complexities of CHM prescriptions often obscure the underlying mechanisms. Our study aims to assess the efficacy of oral CHM in treating CP, a meta-analysis will be conducted on randomized clinical trials (RCTs).

**Materials and methods:**

We searched Cochrane Library, PubMed, Embase, Scopus, PubMed Central, ClinicalTrials.gov, and China National Knowledge Infrastructure (CNKI), from 1990 to 2022. The primary outcome was the improvement in Effectiveness rate (ER). The secondary outcome was the improvement of motor function (GMFM). Subgroup analysis and trial sequential analysis (TSA) were conducted to confirm results consistency. Core CHMs were investigated through system pharmacology analysis.

**Results:**

Seventeen RCTs were analyzed, in which CHMs with Standard treatment (ST) were compared to ST alone. All participants were aged <11 years. More participants in the CHM group achieved prominent improvement in ER (RR: 1.21, 95% CI: 1.13–1.30, *p*-value < 0.001, I^2^ = 32%) and higher GMFM improvement (SMD: 1.49; 95% CI: 1.33–1.65, p-value < 0.001, I^2^ = 92%). TSA also showed similar results with proper statistical power. Core CHMs, such as *Glycyrrhiza uralensis* Fisch. Ex DC., *Poria cocos* (Schw.) Wolf, *Paeonia lactiflora* Pall., processed *Rehmannia glutinosa* (Gaertn.) DC., *Astragalus mongholicus* Bunge, and *Angelica sinensis* (Oliv.) Diels, exerted effects on immune modulation and metabolism systems. The subgroup analysis showed participants using core CHMs or longer CHM treatment duration, and studies enrolling CP with spastic or mixed type, or mild-to-moderate severity had better outcomes in CHM groups with less heterogeneity.

**Conclusion:**

CHMs may have a positive impact on managing pediatric CP; however, the potential bias in study design should be improved.

**Systematic Review Registration:**

Identifier CRD42023424754.

## 1 Introduction

Cerebral palsy (CP) is the most common cause of disability in childhood, with an estimated global prevalence between 0.16% and 0.37% (McIntyre et al., 2022). The term refers to a group of neurological disorders that affect movement and posture along the lifespan, caused by damage to the developing brain. It results in motor disability, and some patients may also develop epilepsy or disturbance of cognition, behavior, communication, sensation, and perception (Rosenbaum et al., 2007; Novak et al., 2012). In terms of socioeconomic aspects, individuals with CP face the impact of multiple disabilities; consequently, they require long-term medication, rehabilitation, and care. Their necessary expenses are significantly higher compared with those of their healthy age-matched counterparts, thus imposing substantial burdens on caregiving families (Vadivelan et al., 2020).

The treatment of CP focuses on improving movement and reducing the disruptions caused to daily activities (Colver et al., 2014). Therefore, physical rehabilitation is currently the standard first-line therapy for CP (Demont et al., 2022). Other therapies include medication, speech rehabilitation, occupational rehabilitation, and surgical intervention (Vitrikas, Dalton, and Breish, 2020). However, recent research indicates that the improvement in gross motor skills through rehabilitation remains limited (Liang et al., 2021). Recent advances in treatment strategies, such as robot-assisted devices and virtual reality, have been used for motor learning and cortical reorganization; nevertheless, the efficacy of these approaches remains uncertain (Bekteshi et al., 2023; Paul et al., 2022).

Consequently, there is a growing interest in exploring alternative medical therapies for improving functional ability and independence of patients with CP. Traditional Chinese medicine (TCM) has been commonly used for centuries as adjunctive therapy in Asia. Studies have found that combining TCM with Standard treatment (ST) can improve motor function and activities of daily living in patients with CP (Zhang et al., 2010; Liao et al., 2017). Moreover, a recent systematic review demonstrated that the combination of TCM and modern rehabilitation therapies may resulted in effective improvements in gross motor function, muscle tone, and functional independence in children with CP (Chen et al., 2023). Thereby, TCM seems to enhance the independence of patients’ daily activity and may reduce the burden on caregivers and the healthcare system. However, previous review articles on TCM interventions often encompassed oral Chinese herbal medicine (CHM), acupuncture, massage, or low-level laser therapy, whereas studies focusing exclusively on CHM remain relatively scarce.

As to CHM efficacy on CP, a recent study utilizing network pharmacology and bioinformatics has elucidated the therapeutic potential of Liuwei Dihuang pills, a traditional CHM, in the treatment and management of CP. The key bioactive constituents of Liuwei Dihuang pills, including quercetin, stigmasterol, and kaempferol, exert their effects of modulating immunological and inflammatory responses through the regulation of several critical signaling pathways, including the PI3K-Akt, IL-17, Jak-STAT, and NF-κB pathways, which are integral to the pathophysiology of CP (Wang et al., 2024). Additionally, in animal study, tanshinone IIA, ingredient of *Salvia miltiorrhiza* Bunge, showed neuroprotective effect and weakened spasticity through inflammation, p38MAPK and VEGF pathway (Zhang et al., 2018). Moreover, a review article reported improved daily activity outcomes when Oriental herbal medicine was integrated into rehabilitation programs (Lee et al., 2018). However, there is still a lack of extensive and up-to-date literature, robust bias assessment, and statistical analysis regarding the efficacy and safety of oral CHMs as well as the core CHMs for CP.

The aim of this study was to compile evidence from recent Randomized clinical trials (RCT) on the use of oral CHM for pediatric CP and assess its potential effectiveness. Additionally, network pharmacology analysis was also undertaken to identify core CHMs utilized in the examined trials and elucidate potential pharmacological pathways involved.

## 2 Materials and methods

This study protocol was prospectively registered in PROSPERO (No. CRD42023424754).

### 2.1 Eligibility criteria

The inclusion criteria were as follows:

1) RCT studies.
2) CP diagnosis was based on diagnostic criteria evaluated by a physician.
3) Age < 18 years.
4) Interventions involved the oral administration of single or mixed traditional CHMs.
5) No limitations based on ethnicity, age, or language.

The exclusion criteria were as follows:

1) Non-RCT studies.
2) Use of folk medicine or traditional medicine other than CHM (i.e., acupuncture, or massage).
3) Studies evaluating the effectiveness of CHMs administered topically (i.e., moxibustion, herbal bath, fumigation therapy).
4) Lack of a control group.
5) The control group did not receive ST.
6) The intervention group did not receive CHM combined with ST.
7) Outcome assessment other than Effectiveness rate (ER), Gross Motor Function Measure score (GMFM), Activities of Daily Living for CP recover evaluation (ADL) (Shuchun, 2000; Yingyuan, 2009), and Modified Ashworth Scale (MAS) score.
8) Studies not published in peer-reviewed journals.
9) Lack of search strategy and information sources.

We conducted thorough searches in various electronic databases from 1 January 1990 to December 2022. The databases included Cochrane Library, PubMed, Embase, Scopus, PubMed Central, ClinicalTrials.gov, and China National Knowledge Infrastructure (CNKI). The specific search approaches are provided in Supplementary Appendix S1. The search terms were used as follow: “Cerebral Palsy” (MeSH Terms) for patient group, and [“Medicine, Chinese Traditional” (Mesh) or “Herbal Medicine” (Mesh) or “Medicine, Korean Traditional” (Mesh) or “Medicine, Kampo” (Mesh)] for intervention.

### 2.2 Data extraction

Huang independently extracted data using a predefined format, as outlined in Table 1, which includes details on the study authors, publication year, sample size, sex, age, intervention, and primary outcomes. Any discrepancies were resolved via deliberations with Cheng, Yang, and Chen. The extracted information included the publication year, study country, study design, CHM content and duration, type of standard management, diagnostic criteria, sample size, participant age and sex, and outcome assessments. Additionally, information regarding interventions, including composition, dosage, and frequency of usage for both control and intervention groups, was recorded. If necessary, and at the discretion of the reviewing author, the corresponding authors of the clinical studies were contacted to obtain any missing data.

**TABLE 1:** Characteristics of included RCTs.

References	Sample size, n (T/C)	Sex, n M:F (T)	Sex, n M:F (C)	Age, mean ± SD (T)	Age, mean ± SD (C)	Type of CP	Treatment intervention (T)	Compare intervention (C)	Intervention formula	Number of compositions in formula	Frequency and duration	Primary outcome
Wu J et al. (2022)	30/30	18:12	19:11	43.07 ± 10.01 months	42.73 ± 10.04 months	Dyskinetic	CHM, PT, OT, speech training, music therapy	PT, OT, speech training, music therapy	Liuwei Dihuang pill and Yigong powder (granule)	10	Unknown frequency for 1 month	GMFM-88, WeeFIM, Gesell
Tung (2022)	45/45	23:22	24:21	4.2 ± 1.5 years	4.1 ± 1.3 years	Spastic	CHM, PT	PT	Huangqi Guizhi Wuwu Tang (decocting pieces)	14	1 dose/day for 30 days	TCM symptom score, ADL, FAC, MWS, 6MWT
Zhang (2021)	36/35	18:18	17:18	34.65 ± 3.10 months	34.36 ± 3.28 months	Mixed	CHM, WM, massage	WM, massage	Kaiqiao Xingshen Decoction (decocting pieces)	7	1 dose/day for 1 month	TCM symptom score, FDA, brain Doppler ultrasound, ER (TDS)
Cai (2020)	42/42	24:18	22:20	3.1 ± 0.8 years	3.3 ± 0.5 years	Spastic	CHM, FES	FES	Huangqi Guizhi Wuwu Tang (decocting pieces)	13	1 dose/day for 8 weeks	GMFM-88, PDMS-2, ER, MAS
Zhang Y et al. (2019)	42/42	23:19	26:16	3.21 ± 0.16 years	3.34 ± 0.18 years	Spastic	CHM, WM, PT	WM, PT	Huangqi Guizhi Wuwu Tang (decocting pieces)	13	1 dose/day, and 4 weeks/course for 3 courses	GMFM-88, TCM symptom score, MAS, PDMS-2, PedsQL, serum BDNF, serum NSE, ER
Geng (2019)	51/51	26:25	27:24	7.41 ± 2.71 years	7.38 ± 2.68 years	Mixed	CHM, WM, acupuncture	WM, acupuncture	Xingnao Kaiqiao Tang (decocting pieces)	14	1 dose/day for 3 months	ER, ADL, FMA PDMS, Berg, MDI, PDI
Liu and Dong (2019)	74/74	44:30	41:33	28.65 ± 12.07 months	28.75 ± 13.12 months	None recorded	CHM, WM, PT, massage	WM, PT, massage	Kaiqiao Xingshen Decoction (decocting pieces)	7	1 dose/day for 1 month	FDA, GMFM, FMFM, ER, serum NSE, serum ET-1, serum IGF-1
Ma et al. (2018)	36/36	24:10	20:11	25.9 ± 18.3 months	25.7 ± 13.4 months	Spastic	CHM, PT, OT, massage, acupuncture, steam therapy	PT, OT, massage, acupuncture, steam therapy	Pujin Keli (granule)	4	≤4 years: 1 dose/day; 4–6 years: 2 doses/day 4 weeks/course for 3 courses	GMFM, Gesell, MAS, TCM symptom score, ER
Sun et al. (2017)	60/60	32:28	33:27	3.38 ± 2.01 years	3.51 ± 2.17 years	Dystonia	CHM, PT	C1-healthy children: no intervention C2-CP: PT	Xingnao Yizhi Fang (decocting pieces)	11	1 dose/day, 10 times/course, 2 days off between courses for 1 year	GMFM-88, serum BDNF, serum TGF-β1, Manual Muscle Testing
Shan et al. (2017)	34/34	24:13	21:10	2.5 ± 2.2 years	2.6 ± 2.1 years	None recorded	CHM, PT, OT, massage, acupuncture, speech training, music therapy, wax therapy, medicinal baths	PT, OT, massage, acupuncture, speech training, music therapy, wax therapy, medicinal baths	Nourishing Kidney and Inducing Resuscitation for Expelling Phlegm Prescription (granule)	10	1.5–3 years, 2/3 pack/day; 4–6 years: 1 pack/day for 4 months	Gesell, ER
Yu et al. (2016)	40/40	21:19	23:17	6.6 ± 3.4 years	6.4 ± 3.2 years	Spastic	CHM, PT, massage	PT, massage	Huangqi Guizhi Wuwu Tang (decocting pieces)	13	1 dose/day for 4 weeks	ER, FAC, MWS, 6MWT
Cheng et al. (2016)	17/13	10:7	8:5	14 months	13 months	None recorded	CHM, PT	PT	High dose of *Astragalus mongholicus* (decocting pieces)	5	1 dose/2 days, and 14 days/course for 10 courses	GMFM, ER
Du et al. (2016)	32/30	20:12	20:10	10.46 ± 3.54 months	9.96 ± 4.18 months	None recorded	CHM	WM	Modified Suanzaoren (granule)	8	1 dose/day for 2 weeks	ER (sleep quality)
Lou and Shi (2016)	30/30	19:11	18:13	2.5 ± 1.3 years	2.6 ± 1.4 years	Spastic	CHM, WM	WM	Shujinhuoluo Wan (pill)	13	1 dose/day, and 6 weeks/course for 10 courses	GMFM, MAS, ADL, WISC, serum IL4, serum IFN-γ, serum IFN-α
Lu et al. (2012)	40/40	25:15	23:17	4.50 ± 1.08 years	4.30 ± 0.79 years	Spastic	CHM, WM, PT	WM, PT	Shenluqizhi Decoction (decocting pieces)	11	1 dose/day and 3 months/course for 2 courses	ER, TCM symptom score, ADL, MAS
Shi and Xie (2015)	70/70	45:25	35:35	7.5 ± 1.5 years	7.8 ± 1.4 years	Spastic	CHM, SPR, WM	SPR, WM	BuShen JianNao (capsule)	9	4 doses/time, 3 times/day for 1.5 months	GMFM-88, ER
Qian (2009)	35/35	24:11	22:13	3.37 ± 0.28 years	3.20 ± 0.36 years	Mixed	CHM, PT	PT	Sijunzi Decoction (decocting pieces)	4	1 dose/day <5 years: tapered for 3 months	ER, saliva amylase amount, serum Zn, serum Fe, hemoglobin

6MWT, 6-min walking test; BDNF, brain-derived neurotrophic factor; Berg, Berg Balance Scale; ET-1, endothelin-1; FAC, functional ambulation category scale; FDA, Frenchay dysarthria assessment; FES, functional electrical stimulation; FMA, Fugl–Meyer assessment scale; FMFM, fine motor function measur; IFN-α, interferon-α; IFN-γ, interferon-γ; IGF-1, insulin-like growth factor 1; IL4, interleukin 4; M:F, male:female; MDI, mental developmental index; MWS, maximum walking speed; NSE, neuron specific enolase; PDI, psychomotor development index; PedsQL, pediatric quality of life inventory; SD, standard deviation; SPR, selective posterior rhizotomy; TDS, Teacher’s Drooling Scale; TGF-β1, transforming growth factor-beta 1; WeeFIM, wee functional independence measure for children; WISC, Wechsler intelligence scale for children.

### 2.3 Quality assessment

Huang and Cheng evaluated the methodological quality using the Risk-of-bias (RoB) assessment tool established by the Cochrane Collaboration (Higgins et al., 2011). Any discrepancies in the assessment were resolved through consultations with Yang and Chen.

### 2.4 Outcome measurements

The primary outcome was the percentage of participants in whom the treatment showed prominent effectiveness. ER was selected since it was commonly used in most studies and provided a composite outcome for participants. It was commonly presented by classifying the clinical response at the end of the study into three grades, such as prominent effectiveness, effectiveness, and ineffectiveness. Prominent improvement, including prominent effectiveness and effectiveness, was confirmed according to the following criteria varied according to different RCTs: 1) GMFM total score improved by ≥1% (Cheng et al., 2016); 2) ≥1/3 symptoms improved (Geng, 2019; Yu, 2016; Lu et al., 2012); 3) TCM syndrome score improved by ≥20% (Zhang L. Q. et al., 2019; Ma et al., 2018); 4) efficacy index improved by ≥1% (Liu and Dong, 2019); 5) drooling improved by ≥1 level (Zhang, 2021); 6) MAS score decreased by ≥1 grade (Shi and Xie, 2015); 7) Peabody developmental motor scale-2 (PDMS-2) improved by ≥1% (Cai, 2020); 8) sleep quality significantly improved (Du et al., 2016); and 9) 10 sports function score improved ≥10 (Qian, 2009). The percentage of prominent improvement was compared between the CHM + ST and ST groups, and this information was extracted as the primary outcome. The secondary outcome included improvement of solely evaluated clinical score systems, such as the Gesell Developmental Scale (Gesell), GMFM indicating motor function, ADL, and MAS presenting the severity of limb spasticity.

### 2.5 Statistical analysis

The analysis of all data was conducted utilizing Cochrane Review Manager 5.4.1. (The Cochrane Collaboration, 2014). The proportion of participants with prominent improvement in ER was analyzed using the Risk ratio (RR) and a 95% Confidence interval (CI). Numerical outcomes were analyzed using the Standardized mean difference (SMD) and/or Mean difference (MD). For data synthesis, a random-effects model with the Mantel–Haenszel test was used to summarize inverse variance and dichotomous data for continuous data. Heterogeneity between the studies was assessed using the I^2^-statistic. A funnel plot was used to detect publication bias. If bias was present, the trim and fill method (Peters et al., 2007) would be applied for correction. Additionally, Trial sequential analysis (TSA) was performed to confirm the efficacy of CHM. TSA is a novel method for evaluating treatment efficacy through interventional meta-analysis study in a more robust manner to mimic large-scale clinical trials (Wetterslev, Jakobsen, and Gluud, 2017; De Cassai et al., 2021; Kang, 2021). In this study, we adopted 5% type I error with 90% power of statistical examination in TSA to evaluate the consistency of results and the adequacy of the number of cases. TSA was carried out using proprietary software (Lan and DeMets, 1983). A system pharmacology analysis was conducted on the prescriptions from the included studies. Detailed methodologies are outlined in Supplementary Appendix S2. In summary, the Chinese herbal medicine network (CMN) was employed to identify the core CHMs, illustrating graphically the commonly used CHMs for CP. The pharmacological pathways of these core CHMs were clarified by referencing online databases for pharmacology pathways. Utilizing this well-established approach, we previously compared the effectiveness of CHM versus Western medicine (WM) in managing Coronavirus disease 2019 (COVID-19), allergic diseases, and diabetic nephropathy (Lu et al., 2022; Chen et al., 2015; Wu et al., 2021; Wang et al., 2023).

We conducted four subgroup analyses. Firstly, based on the type of CP, we divided the participants into spastic and mixed types. Secondly, dividing different initial symptom severity into three groups due to CP baseline severity was a main influencing factor to prognosticate long-term functional outcome. We used the ADL, Gesell, and MAS scores for categorization (mild, ADL: ≥91, Gesell: ≥55, MAS: <2; moderate, ADL: 61–90, Gesell: 40–54, MAS: 2; and severe, ADL: ≤60, Gesell: ≤39, MAS: >2) (Yuan et al., 2021; Huifang, 2012; Winstein et al., 2016). We divided the subgroup into mild-to-moderate and severe. Thirdly, we used the duration of the treatment course. We divided the duration into three subgroups, namely, 0–1 month, 1–3 months, and 3–6 months. We selected 1 month as the first cut point due to the shortest period for observing the efficacy of CHM (Yoo et al., 2016). Three months was the fastest time for neural recovery (Boecker et al., 2022; Schalow, 2002), and 6 months represented chronic phase of recovery (Gao et al., 2022). Finally, we extracted the studies that utilized core CHMs. For all analyses, excluding TSA, *p*-values < 0.05 denoted statistical significance.

## 3 Results

### 3.1 Literature search

Our electronic and manual searches yielded a total of 161 references. After removing two duplicate records, 159 studies remained. A detailed examination of titles and abstracts led to the exclusion of 88 studies.

**FIGURE 1:**
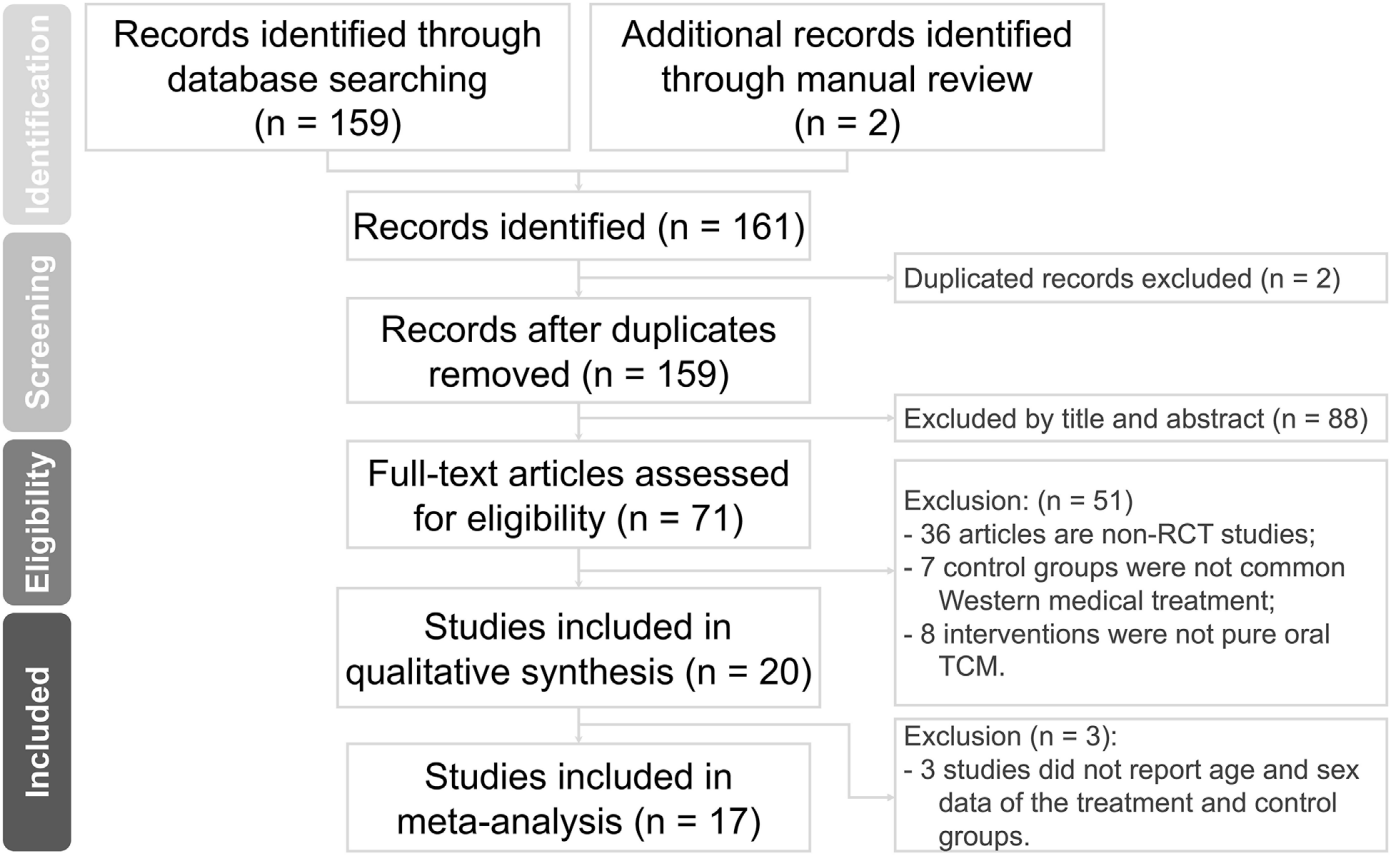
Flowchart of the search strategy.

After this initial screening, we proceeded to retrieve and carefully evaluate the complete texts of 71 references. Based on the inclusion and exclusion criteria, 51 studies were removed. Furthermore, three studies were excluded due to the lack of detailed data on the CHM and ST groups. Finally, our comprehensive assessment led to the inclusion of 17 studies, involving a total of 1,421 participants. These data are illustrated in Figure 1.

### 3.2 Description of included studies

#### 3.2.1 Characteristics of studies

Table 1 shows the detailed information of the analyzed studies, which were all RCTs. Sixteen studies adopted a two-arm parallel design, and only one used a three-arm design, in which only data from CHM and the control arm were extracted. All selected studies were sourced from China.

#### 3.2.2 Characteristics of participants

In the analyzed studies, the age of the participants ranged from 0 to 11 years old. In terms of diagnostic criteria and classification, 11 studies followed the Chinese national clinical diagnostic criteria and classification as their standard, whereas six studies referred to the Rehabilitation Guideline for CP in China. Regarding the type of CP, eight studies enrolled only patients with the spastic type, while the remaining enrolled participants with all types of CP.

#### 3.2.3 Design of the control group

ST, including Physical therapy (PT) and Occupational therapy (OT), was found in the control group of 11 trials. Five trials only used WM, and three trials used rehabilitation plus WM in the control group. The WM prescribed in trials included baclofen, dantrolene sodium, midazolam, phenobarbital, cerebrolysin, ligustrazine hydrochloride, or other medicines for nourishing neurons. With regard to ST, five trials added massage, and some added complementary therapy, such as speech training, dry needle therapy, steam therapy, music therapy, wax therapy, and medicinal baths. Notably, one study used Functional electrical stimulation (FES) in the control group, while another used Selective posterior rhizotomy (SPR) plus WM. The disparities among the experimental herbal formulas combined with PT, PT alone, and no treatment (i.e., healthy children) were discussed in a three-arm parallel trial.

#### 3.2.4 Design of the intervention group

All included trials involved a combination of CHM with ST, and all prescriptions were mixed CHMs. The number of CHMs used in trials ranged from 4 to 14 (mean: 9; SD: 3). The frequency of CHMs combination usage was shown in Supplementary Appendix S3. Among all CHMs, *Glycyrrhiza uralensis* Fisch. ex DC. (GU) and *Poria cocos* (Schw.) Wolf. (PC) are the most frequently used combination of medications (47.059%). The duration of treatment ranged from 2 weeks to 15 months.

### 3.3 Quality of trials

**FIGURE 2:**
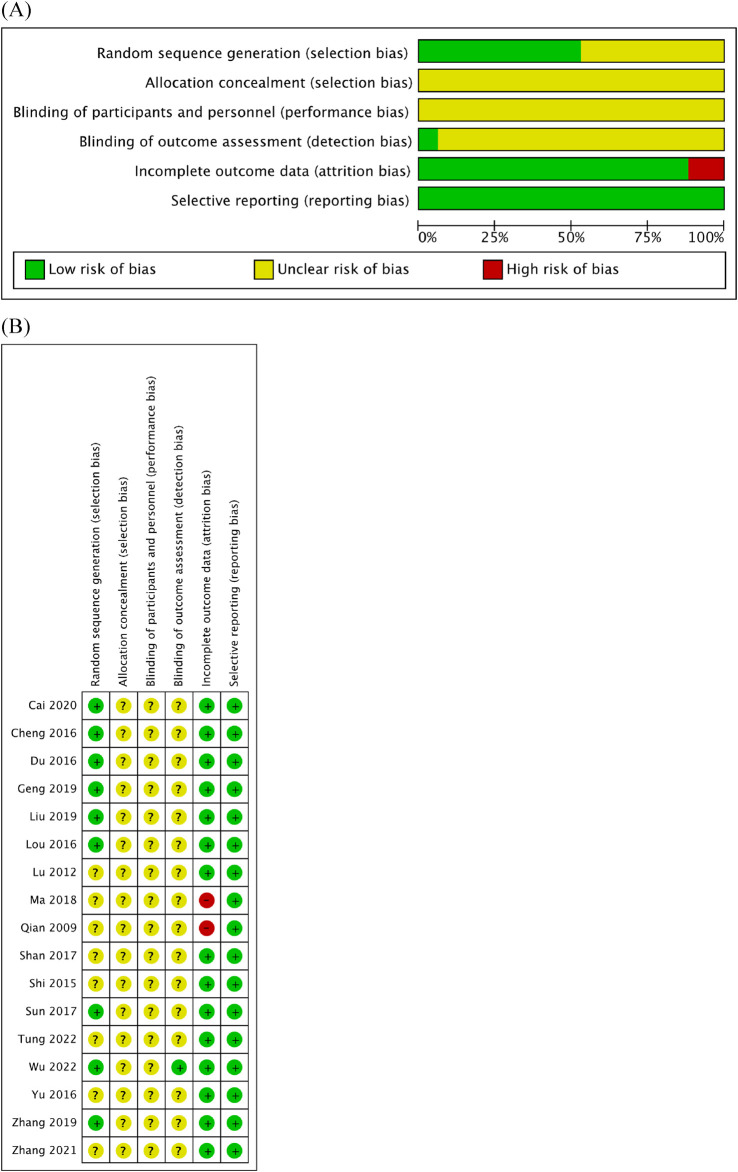
Quality assessment of 17 included studies using the RoB assessment tool established by the Cochrane Collaboration. **(A)** RoB graph. **(B)** RoB summary.

Quality assessment was performed using Cochrane RoB (Figures 2A, B). Within the RoB assessment, most studies displayed unclear statuses of allocation bias, performance bias, and detection bias. Evaluation of the selection bias indicated that nine of the RCTs included in this analysis were at a low RoB, while the status of others remained unclear. Similarly, the risk of attrition bias was low across the majority of RCTs, except for two studies that were linked to high risk. Notably, all studies were rated as having a low risk of reporting bias.

### 3.4 Meta-analysis of included studies

#### 3.4.1 Primary outcome: the RR of achieving prominent improvement in ER

**FIGURE 3:**
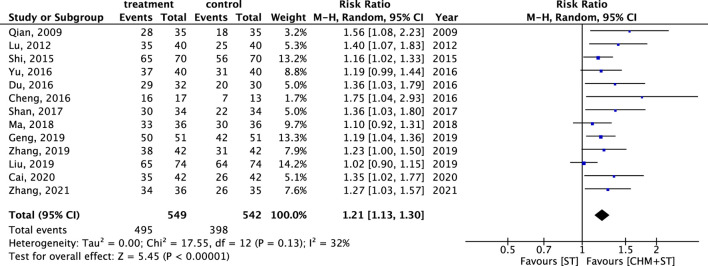
Forest plot of meta-analysis comparing CHM + ST with ST in terms of ER.

Generally, the CHM + ST group had better outcomes than the ST group. In 13 RCTs analyzed, the CHM + ST group had a superior proportion of participants with prominent improvement (495/549, 90.16%) compared with the ST group (398/542, 73.43%). The CHM + ST group demonstrated a 21% higher proportion of prominent improvement compared with the ST groups (RR: 1.21, 95% CI: 1.13–1.30, p-value < 0.001, I^2^ = 32%) (Figure 3). Moreover, the TSA confirmed this result, and the total pooled case number (n = 1,091) achieved the threshold of 90% statistical examination power (n = 310) (Supplementary Appendix S4).

#### 3.4.2 Secondary outcome: improvement of Gesell, GMFM, ADL, and MAS scores

**FIGURE 4:**
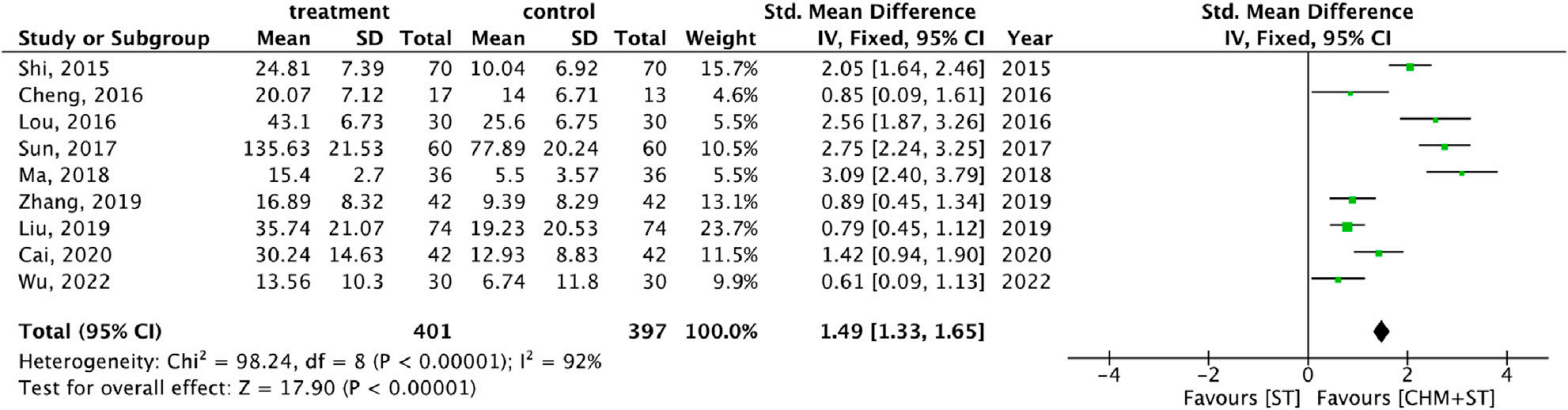
Forest plot of meta-analysis comparing CHM + ST with ST in terms of improvement of GMFM score change from the baseline.

**FIGURE 5:**
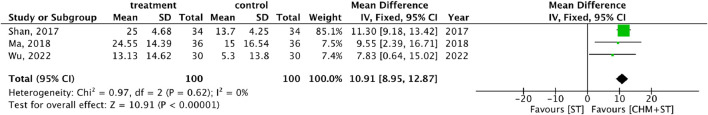
Meta-analysis comparing CHM + ST with ST in terms of Gesell Developmental Schedule improvement from baseline.

**FIGURE 6:**
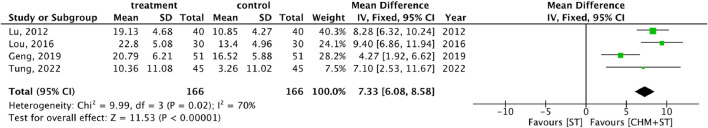
Meta-analysis comparing CHM + ST with ST in terms of improvement of ADL score from baseline.

**FIGURE 7:**
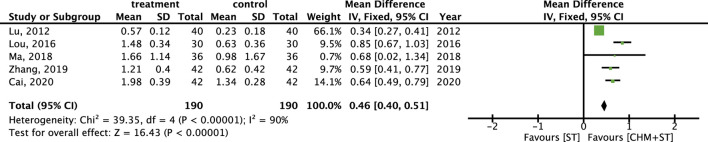
Meta-analysis comparing CHM + ST with ST in terms of improvement of MAS level from baseline.

More than half of the studies used GMFM (n = 9) to measure motor function disorder. The mean of improvement of GMFM scores of the intervention and control groups ranged from 13.56 (minimum)–135.63 (maximum) and 5.5 (minimum)–77.89 (maximum), respectively. The pooled analysis revealed a significantly better improvement in the GMFM score in the CHM + ST group versus the ST group (SMD: 1.49; 95% CI: 1.33–1.65, *p*-value < 0.001, I^2^ = 92%) (Figure 4). Three RCTs were included in the Gesell analysis. The CHM + ST group exhibited a more significant change in scores compared to the ST group (MD: 10.91; 95% CI: 8.95–12.87, *p*-value < 0.001, I^2^ = 0%) (Figure 5). Four studies reported the improvement of daily living function using the ADL score. Greater ADL improvement was noted in the CHM + ST group compared with the ST group (MD: 7.33; 95% CI: 6.08–8.58, p-value < 0.001, I^2^ = 70%) (Figure 6). Five studies were included in the MAS analysis. Greater MAS improvement was recorded in the CHM + ST group versus the ST group (MD: 0.46; 95% CI: 0.40–0.51, p-value < 0.001, I^2^ = 90%) (Figure 7).

### 3.5 Subgroup meta-analysis

**FIGURE 8:**
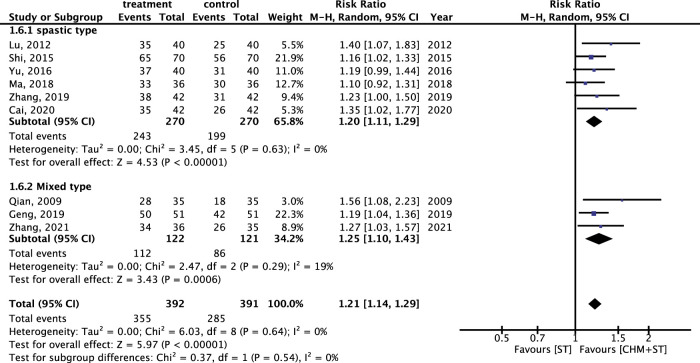
Subgroup analysis of RCTs. Comparison of spastic type with mixed type CP based on improvement in the ER.

Within different subtypes of CP, durations, and degrees of severity, the subgroup analysis showed lower heterogeneity with consistent results. For both spastic (n = 540) and mixed types (n = 243) of CP, the CHM + ST group had a significantly higher proportion of prominent improvement in ER compared with the ST group (RR: 1.20, 95% CI: 1.11–1.29, *p*-value < 0.001, I^2^ = 0% vs. RR: 1.25, 95% CI: 1.10–1.43, *p*-value < 0.001, I^2^ = 19%) (Figure 8).

**FIGURE 9:**
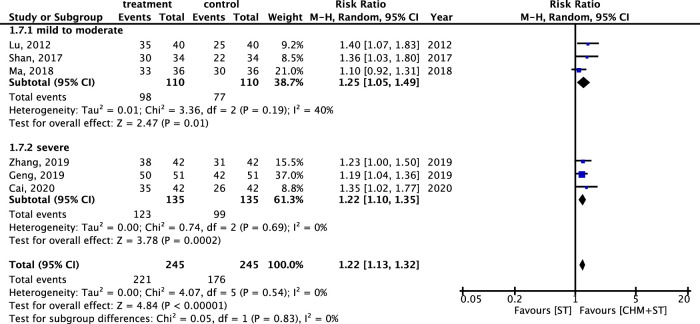
Meta-analysis of ER under subgroups for different degrees of CP severity.

The RR of ER within different degrees of CP severity was also analyzed. Six studies were analyzed after screening. In both categories, there was a notable enhancement in ER in the CHM + ST group versus the ST group. In addition, the mild-to-moderate subgroup (n = 220, RR: 1.25, 95% CI: 1.05–1.49, *p*-value = 0.001, I^2^ = 40%) exhibited better improvement than the severe subgroup (n = 270, RR: 1.22, 95% CI: 1.10–1.35, *p*-value < 0.001, I^2^ = 0%) (Figure 9).

**FIGURE 10:**
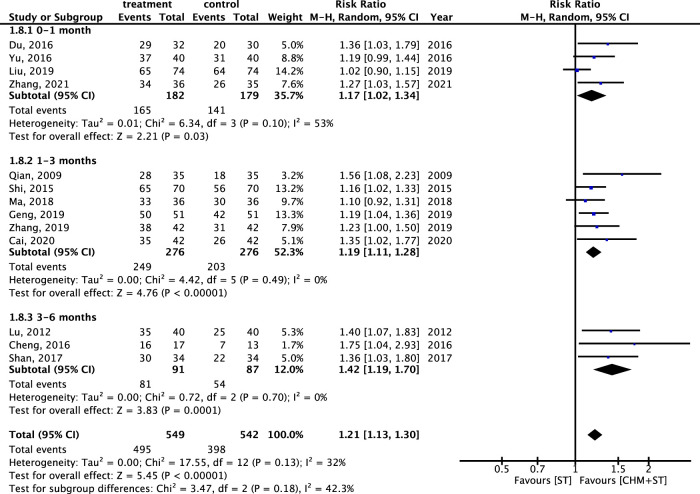
Meta-analysis of ER under subgroups for different durations of treatment.

Furthermore, regarding the duration of the treatment course, the CHM + ST group also showed better results compared with the ST group. Patients receiving treatment for 3–6 months (n = 178, RR: 1.42, 95% CI: 1.19–1.70, *p*-value < 0.001, I^2^ = 0%) showed the greatest improvement, followed by those treated for 1–3 months (n = 552, RR: 1.19, 95% CI: 1.11–1.28, *p*-value < 0.001, I^2^ = 0%), and 0–1 month (n = 361, RR: 1.17, 95% CI: 1.02–1.34, *p*-value = 0.03, I^2^ = 53%) (Figure 10).

### 3.6 CMN for CP obtained from the included trials

**FIGURE 11:**
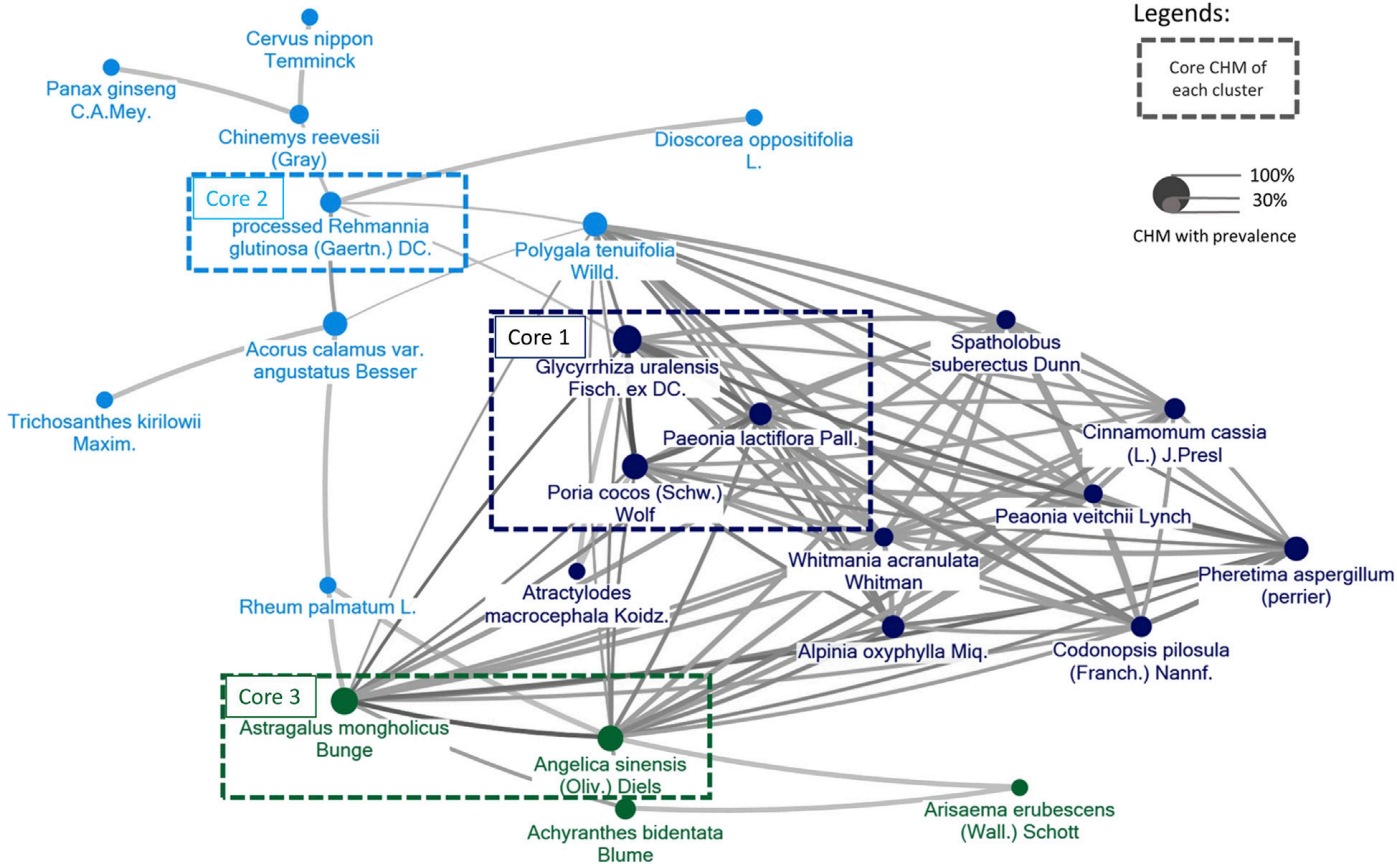
The CMN for CP was derived from the trials included in the study. Colors assigned to each node (agent) represent distinct clusters of CHM, with higher prevalence represented by larger node sizes. Darker colors and thicker connecting lines signify a more pronounced and frequent mixture of two CHMs.

**FIGURE 12:**
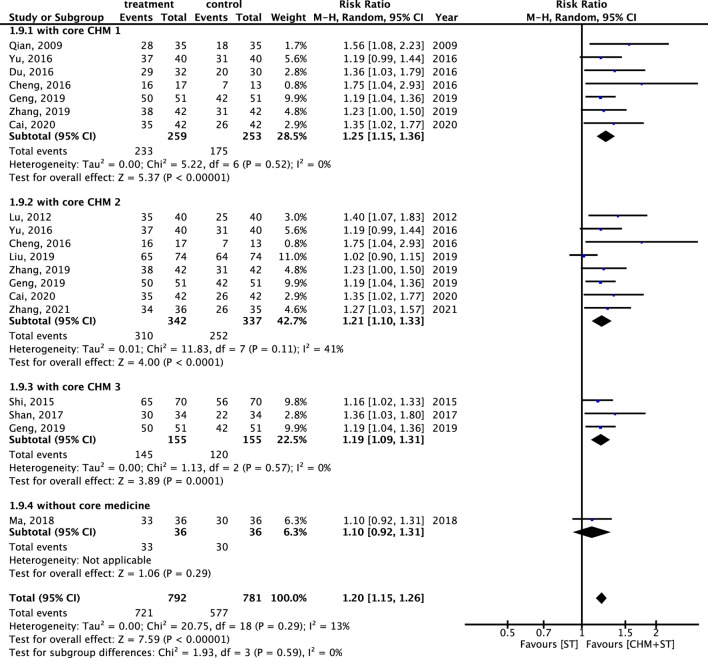
Subgroup analysis of RCTs. Comparison of the effects of three clusters of core medicines on the improvement of ER.

The components of CHMs in each included study were itemized in Supplementary Appendix S5. The prevalence of CHM applied was presented in order in Supplementary Appendix S6. CMN could be constructed based on these CHM connections and present as Figure 11. Among these, three sets of core CHMs were found, i.e., core CHM1: GU, PC, and *Paeonia lactiflora* Pall. (PL); core CHM2: *Rehmannia glutinosa* (Gaertn.) DC. (RG) (present in 29% of all studies); and core CHM3: *Angelica sinensis* (Oliv.) (AS) and *Astragalus mongholicus* Bunge (AM). Compared with CHM + WM that did not include core medicines, CHM + WM including the three aforementioned sets of core CHMs exhibited better effectiveness (core CHM1, n = 512, RR: 1.25, 95% CI: 1.15–1.36, *p*-value < 0.001; core CHM2, n = 679, RR: 1.21, 95% CI: 1.10–1.33, *p*-value < 0.001; core CHM3, n = 310, RR: 1.19, 95% CI: 1.09–1.31, *p*-value < 0.001; without core medicine, n = 72, RR: 1.10, 95% CI: 0.92–1.31, *p*-value = 0.29) (Figure 12).

**FIGURE 13:**
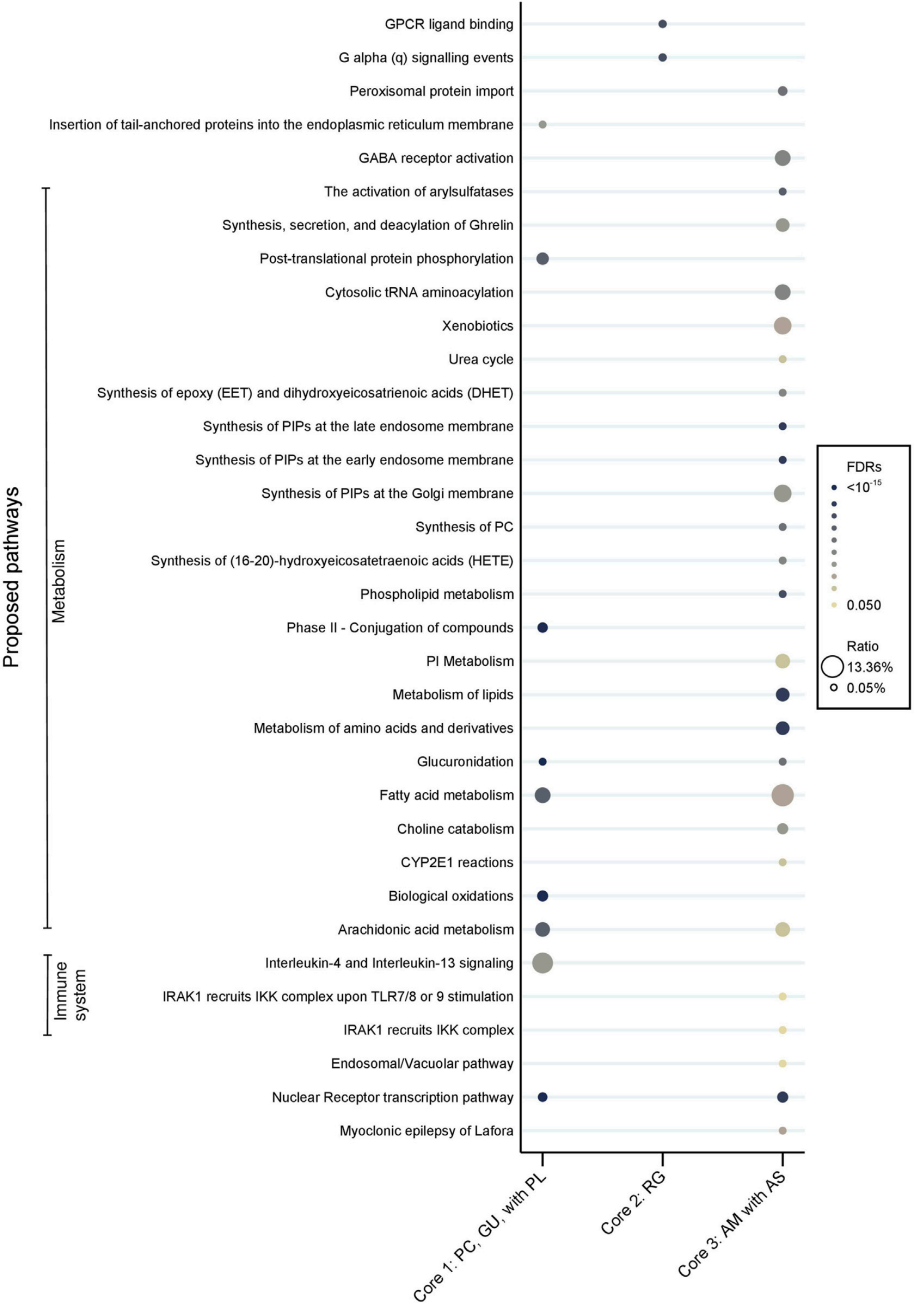
Pharmacologic pathways of CHM. Core 1: PC, GU, with PL; Core 2: RG; Core 3: AM with AS. (PC, *Poria cocos* (Schw.) Wolf; GU, *Glycyrrhiza uralensis* Fisch. ex DC.; PL, *Paeonia lactiflora* Pall.; RG, processed *Rehmannia glutinosa* (Gaertn.) DC.; AM, *Astragalus mongholicus* Bunge; AS, *Angelica sinensis* (Oliv.) Diels.

Moreover, noticeable disparities were observed in the proposed pharmacological pathways between the core CHMs (Figure 13). In terms of the immune system, core CHM1 (PC, GU, and PL) acted on Interleukin 4 (IL4) and Interleukin 13 (IL13) signaling. Additionally, core CHM3 (AS and AM) played a crucial role in modulating the activation of the γ-aminobutyric acid (GABA) receptor. Moreover, with regard to metabolism pathways, cores CHM1 and CHM3 demonstrated multiple advantages, particularly in aspects of arachidonic acid metabolism.

### 3.7 Publication bias

The funnel plots exhibited a low risk of publication bias (Supplementary Appendix S7). Moreover, the corrected results using Trim and Fill approach remained significant (Effective size 1.170, 95% CI: 1.084–1.256) (Supplementary Appendix S8).

### 3.8 Adverse drug events (ADEs) of CHM

Of the 17 included studies, only five reported side effects. In one study, side effects were only observed in the control group. The remaining four studies described that the patients treated with CHM + ST experienced side effects, such as gastrointestinal discomfort (i.e., nausea, vomiting, or diarrhea), although of no significance in comparison to the ST group. Moreover, there were no significant changes in liver and renal function in the CHM groups.

## 4 Discussion

To the best of our knowledge, this is the first meta-analysis for pediatric CP involving core CHM exploration and TSA. In all studies, we found the use of oral CHM in combine with ST led to a significantly higher proportion of patients achieving prominent improvement in ER compared with control. Improvements in motor skills, developmental status, self-care abilities, and muscle rigidity were consistently observed. In addition, the ER was higher for CHM + ST versus ST regardless of the type or severity of CP. Additionally, our meta-analysis showed that a longer duration of treatment is associated with better results. Regarding prescriptions, various types of CHM were used in the studies as other meta-analyses of CHMs (Wieland et al., 2013; Chung et al., 2015). Through the CMN, it was possible to efficiently identify the potential core CHMs for pediatric CP.

Recent advances in the treatment of CP include numerous methods, such as hyperbaric oxygen (Laureau et al., 2022), stem cell therapy (Novak et al., 2023), virtual reality rehabilitation (Han and Park, 2023), and robot-assisted devices (Vezér et al., 2024; Conner et al., 2022), which are currently under investigation. However, pharmacological options for CP remain limited. For children with CP, early intervention is more beneficial, as it minimizes the potential impact of muscle tension and poor posture on motor skills, thereby preventing hindrances in daily activities (Bobath, 1967). Therefore, CHM could be used as a complementary therapy with a good safety profile. Additionally, according to our results, incorporating the use of CHM for 1 month can lead to noticeable improvements in CP syndromes. Moreover, continuing combined therapy for 3–6 months seems appropriate, as supported by the subgroup analysis conducted in this study. CHMs might not only have the effect of muscle relax like WM, but also the effect of motor function and developmental status improvement.

CP is caused by disturbance or injury to the developing brain, often as a consequence of hypoxia, infection, stroke, or hypotension; the subsequent inflammatory cascade follows the original insult (Wimalasundera and Stevenson, 2016). Recent studies found higher levels of inflammatory markers in infants and children with CP, which might have a relationship between inflammation and neural damage at the perinatal period and during development of children (Paton et al., 2022; Magalhães et al., 2019; Malaeb and Dammann, 2009; Bashiri et al., 2006). Increased of cytokine IL-4 and IL-13 in CP patient were mentioned in some studies and were thought to have relationship with neural injury (Than et al., 2023; Djukic et al., 2009; Kaukola et al., 2004). And arachidonic acid, which might activate neuroinflammatory response and overproduction of proinflammatory cytokine, might lead to the serious of white matter damage and CP development as a consequence (Kapitanović Vidak et al., 2017; Chun et al., 2015; Strickland, 2014). Therefore, there is a growing interest in neuroprotective effect in CP through anti-inflammatory agent (Salomon, 2024; Mallah et al., 2020). Based on the current hypothesis and our findings on the anti-inflammatory effects of CHM, core CHM1 (PC, PL, GU) can modulate IL4 and IL13, while cores CHM1 and CHM3 (PC, PL, GU, AS, AM) are related to the arachidonic acid pathway. Previous studies also revealed that all these agents possess anti-inflammatory properties (Li et al., 2022; Wu J et al., 2022; He and Dai, 2011; Li et al., 2020; Gong et al., 2022), which may have benefit in reducing nerve damage caused by inflammation.

As to core CHM2 (RG), catalpol is one of the active ingredients in RG; it exerts a neuroprotective effect against hypoxic/ischemic injury by inhibiting apoptosis and regulating Aquaporin-4 (AQP4) expression (Jiang et al., 2015; Zhang Y et al., 2019). Besides, catalpol can promote angiogenesis via enhancing vascular endothelial growth factor-phosphatidylinositol 3 kinase/protein kinase B (VEGF-PI3K/AKT) and VEGF- Mitogen Activated Protein Kinase Kinase 1/2/extracellular signal-regulated kinase 1/2 (VEGF-MEK1/2/ERK1/2) signaling (Wang et al., 2020; Wang et al., 2022). Moreover, rehmannioside A, which is derived from RG, has neuroprotection effects and improves cognitive impairment by inhibiting ferroptosis and activating the PI3K/AKT/Nuclear factor erythroid 2–related factor 2 (Nrf2) and solute carrier family 7 member 11/glutathione peroxidase 4 (SLC7A11/GPX4) signaling pathway (Fu et al., 2022). Additionally, catalpol and mannitol, which are two components of RG, have anticonvulsant effects via GABA_A_ receptor regulation (Kim et al., 2020).

In our study, we found that core CHM3 (AS and AM) played a crucial role in modulating GABA receptor activation and arachidonic acid metabolism. Previous studies showed that AM and AS upregulated VEGF expression to modulate the function of capillaries (Song et al., 2009). Gelispirolide and riligustilide, which are two phthalide dimmers isolated from AS, exert a GABAergic effect to relax spastic muscle (Deng et al., 2006). Besides, previous studies have demonstrated the anti-inflammatory effects of AS and AM (Li et al., 2020; Gong et al., 2022). Collectively, the available evidence indicates that the combination of CHM with conventional therapy may bring more advantages for patients with CP.

The safety of treatment using CHM was assessed by analyzing reported adverse reactions. Only mild side effects, such as gastrointestinal discomfort (nausea, vomiting, or diarrhea), were reported, without significant differences compared with the ST group. Furthermore, commonly used WM, such as baclofen, may cause central nervous system adverse reactions (e.g., confusion, dizziness, drowsiness, sedation, and asthenia) (Alstermark et al., 2008). Diazepam and clonazepam may be associated with side effects including sedation, cognitive impairment, amnesia, and ataxia (Vinkers and Olivier, 2012). Botulinum toxin injections lead to muscle atrophy and muscle weakness (Kaya Keles and Ates, 2022). Unlike WM, CHM does not cause drowsiness or muscle weakness; furthermore, it does not affect the daily life and rehabilitation schedule of children with CP.

## 5 Limitations

There are some limitations in this study. Firstly, there was a high heterogeneity observed in the results for the GMFM, ADL, and MAS scores. This heterogeneity may be due to the various types of CHM used. Therefore, we presented the core medicine network for CP to simplify the intervention and eliminate the diversity for future clinical trials. Secondly, although selection, attrition, and reporting biases were low, the allocation and performance biases were mostly unclear. Common sources of biases included uncertain concealment, lack of a specific blinding process, and randomization. Consequently, there is a need for high-quality clinical trials with improved designs. Thirdly, the generalizability of the present findings is poor. All studies included in this analysis were conducted in China; hence, the ethnic diversity is limited. These studies did not include participants from Caucasian, African, or Hispanic populations. Fourthly, the sample size in the included trials was comparatively modest (Sakpal, 2010). Therefore, we used TSA to confirm the results in this meta-analysis, which achieved the threshold of 90% statistical examination power. Fifthly, the hypothesis that CHM could improve CP through anti-inflammatory effects remains speculative. There was evidence supporting that CHM had anti-inflammatory properties and that inflammation can be mitigated in CP, but direct evidence is lacking. Therefore, larger CHM related RCTs with inflammatory biomarker analysis was needed to detail the mechanistic aspects and the relationship with CP.

## 6 Conclusion

CHM has the potential to treat pediatric CP in terms of improving motor function, developmental status, daily living function, and spasticity, as well as avoiding the occurrence of serious ADEs. We also identified core medications for treating CP and possible drug action pathways for reference in future clinical use. Subgroup analysis revealed that the combination of CHM with conventional treatment demonstrated better efficacy when core CHMs were included, the treatment duration was extended, or when patients had mild-to-moderate baseline severity. However, the included studies exhibited considerable biases over allocation and performance, high level of heterogeneity, poor generalizability, small sample size in the analysis. Therefore, further rigorous, multicenter, larger, and high-quality research is warranted.

## Data Availability

The original contributions presented in the study are included in the article/Supplementary Material, further inquiries can be directed to the corresponding author.

## Glossary

6MWT: 6-minute walking test
ADEs: Adverse drug events
ADL: Activities of Daily Living for CP recover evaluation
AKT: Protein kinase B
AM: *Astragalus mongholicus* Bunge
AQP4: Aquaporin-4
AS: *Angelica sinensis* (Oliv.)
BDNF: Brain-derived neurotrophic factor
Berg: Berg Balance Scale
CHM: Chinese herbal medicine
CI: Confidence interval
CMN: Chinese herbal medicine network
CNKI: China National Knowledge Infrastructure
COVID-19: Coronavirus disease 2019
CP: Cerebral palsy
ER: Effectiveness rate
ERK1/2: Extracellular signal-regulated kinase 1/2
ET-1: Endothelin-1
FAC: Functional ambulation category scale
FDA: Frenchay Dysarthria Assessment
FES: Functional electrical stimulation
FMA: Fugl–Meyer assessment scale
FMFM: Fine motor function measure
GABA: γ-aminobutyric acid
Gesell: Gesell Developmental Scale
GMFM: Gross Motor Function Measure score
GPX4: Glutathione peroxidase 4
GU: *Glycyrrhiza uralensis* Fisch. ex DC.
IFN-α: Interferon-α
IGF-1: Insulin-like growth factor 1
IL13: Interleukin 13
IL4: Interleukin 4
M:F: Male:Female
MAS: Modified Ashworth Scale
MD: Mean difference
MDI: Mental Developmental Index
MEK1/2: Mitogen Activated Protein Kinase Kinase 1/2
MWS: Maximum walking speed
Nrf2: Nuclear factor erythroid 2–related factor 2
NSE: Neuron specific enolase
OT: Occupational therapy
PC: *Poria cocos* (Schw.) Wolf.
PDI: Psychomotor Development Index
PDMS-2: Peabody developmental motor scale-2
PedsQL: Pediatric Quality of Life Inventory
PI3K: Phosphatidylinositol 3 kinase
PL: *Paeonia lactiflora* Pall.
PT: Physical therapy
RCT: Randomized clinical trial
RG: *Rehmannia glutinosa* (Gaertn.) DC.
RoB: Risk-of-bias
RR: Risk ratio
SD: Standard deviation
SLC7A11: Solute carrier family 7 member 11
SMD: Standardized mean difference
SPR: Selective posterior rhizotomy
ST: Standard treatment
TCM: Traditional Chinese medicine
TDS: Teacher’s Drooling Scale
TGF-β1: Transforming growth factor-beta 1
TSA: Trial sequential analysis
VEGF: Vascular endothelial growth factor
WeeFIM: Wee Functional Independence Measure for Children
WISC: Wechsler Intelligence Scale for Children
WM: Western medicine
