# Supplementary material for: Chinese herbal medicine for the treatment of children with cerebral palsy: a meta-analysis of randomized controlled trials with core herbs exploration
Source: Front Pharmacol. 2025 Feb 26;16:1500095. doi: 10.3389/fphar.2025.1500095 (PMC11897310; doi:10.3389/fphar.2025.1500095)
Supplement: Supplementary file 1 [file DataSheet2.pdf]

Appendix 5. Composition of the formulas used in each study

| Reference | Formula | Composition of formula |
|---|---|---|
| Wu et al. (2022) | Liuwei Dihuang pill and Yigong powder (granule) | Processed *Rehmannia glutinosa* (Gaertn.) DC. 10g, *Cornus officinalis* Siebold & Zucc. 5 g, *Dioscorea oppositifolia* L. 5 g, *Poria cocos* (Schw.) Wolf 5 g, *Alisma plantago-aquatica* L. 5 g, *Paeonia × suffruticosa* Andrews 3 g, *Panax ginseng* C.A. Mey. 6 g, *Atractylodes macrocephala* Koidz. 9 g, *Citrus reticulata* Blanco 6 g, *Glycyrrhiza uralensis* Fisch. ex DC. 3 g; some may add *Chinemys reevesii* (Gray), *Paeonia lactiflora* Pall., *Gastrodia elata* Blume, *Phellodendron chinense* C.K. Schneid., *Curcuma longa* L. |
| Tung et al. (2022) | Huangqi Guizhi Wuwu Tang (decocting pieces) | *Astragalus mongholicus* Bunge 12 g, *Cinnamomum cassia* (L.) J. Presl 9 g, *Spatholobus suberectus* Dunn 9 g, *Codonopsis pilosula* (Franch.) Nannf. 9 g, *Alpinia oxyphylla* Miq. 9 g, *Peaonia veitchii* Lynch 6 g, *Polygala tenuifolia* Willd. 6 g, *Poria cocos* (Schw.) Wolf 6 g, *Angelica sinensis* (Oliv.) Diels 6 g, *Paeonia lactiflora* Pall. 6 g, *Pheretima aspergillum* (perrier) 6 g, *Whitmania acranulata* Whitman 6 g, *Poria cocos* (Schw.) Wolf 6 g, *Glycyrrhiza uralensis* Fisch. ex DC. 6 g; for shoulder, back, or upper limbs pain: add *Saposhnikovia divaricata* (Turcz.) Schisch. 12 g, *Notopterygium incisum* Ting. ex H. T. Chang 12 g; for lower back pain: add *Eucommia ulmoides* Oliv. 15 g; for lower extremity pain: add *Stephania tetrandra* S. Moore 15 g, *Achyranthes bidentata* Blume 15 g |
| Zhang et al. (2021) | Kaiqiao Xingshen Decoction (decocting pieces) | *Astragalus mongholicus* Bunge 10 g, *Achyranthes bidentata* Blume 2 g, *Acorus calamus* var. angustatus Besser 2 g, *Angelica sinensis* (Oliv.) Diels 2 g, *Arisaema erubescens* (Wall.) Schott 5 g, *Trichosanthes kirilowii* Maxim. 3 g, *Rheum palmatum* L. 3 g |
| Cai et al. (2020) | Huangqi Guizhi Wuwu Tang (decocting pieces) | *Astragalus mongholicus* Bunge 12 g, *Cinnamomum cassia* (L.) J. Presl 10 g, *Peaonia veitchii* Lynch 6 g, *Polygala tenuifolia* Willd. 6 g, *Paeonia lactiflora* Pall. 6 g, *Codonopsis pilosula* (Franch.) Nannf. 9 g, *Spatholobus suberectus* Dunn 9 g, *Angelica sinensis* (Oliv.) Diels 6 g, *Pheretima aspergillum* (perrier) 6 g, *Alpinia oxyphylla* |

| | | |
|---|---|---|
| | | Miq. 9 g, *Poria cocos* (Schw.) Wolf 6 g, *Whitmania acranulata* Whitman 6 g, *Glycyrrhiza uralensis* Fisch. ex DC. 6 g |
| Zhang et al. (2019) | Huangqi Guizhi Wuwu Tang (decocting pieces) | *Astragalus mongholicus* Bunge 12 g, *Spatholobus suberectus* Dunn 9 g, *Cinnamomum cassia* (L.) J. Presl 9 g, *Alpinia oxyphylla* Miq. 9 g, *Codonopsis pilosula* (Franch.) Nannf. 9 g, *Angelica sinensis* (Oliv.) Diels 6 g, *Peaonia veitchii* Lynch 6 g, *Pheretima aspergillum* (perrier) 6 g, *Poria cocos* (Schw.) Wolf 6 g, *Whitmania acranulata* Whitman 6 g, *Paeonia lactiflora* Pall. 6 g, *Glycyrrhiza uralensis* Fisch. ex DC. 6 g, *Polygala tenuifolia* Willd. 6 g |
| Geng et al. (2019) | Xingnao Kaiqiao Tang (decocting pieces) | *Astragalus mongholicus* Bunge 20 g, *Pueraria lobata* (Willd.) Ohwi 15 g, *Poria cocos* (Schw.) Wolf 12 g, *Pheretima aspergillum* (perrier) 10 g, *Acorus calamus* var. angustatus Besser 10 g, *Curcuma longa* L. 10 g, *Polygonatum sibiricum* Redoute 10 g, *Rehmannia glutinosa* (Gaertn.) DC. 10 g, processed *Rehmannia glutinosa* (Gaertn.) DC. 10 g, *Conioselinum anthriscoides* 'Chuanxiong' 10 g, *Paeonia lactiflora* Pall. 10 g, *Atractylodes macrocephala* Koidz. 10 g, *Angelica sinensis* (Oliv.) Diels 6 g, *Glycyrrhiza uralensis* Fisch. ex DC. 6 g; for poor digestion: add *Gallus gallus domesticus* Brisson 12 g, *Massa Fermentata* Medicinalis 10 g, *Citrus aurantium* L. 6 g; for constipation: add *Rheum palmatum* L. 6 g, then add Mirabilitum 4 g for severe cases; for chillness and cold limbs: add *Aconitum carmichaeli* Debeaux 6 g, *Cinnamomum cassia* (L.) J. Presl 6 g; for poor sleep quality: add *Ziziphus jujuba* Mill. 10 g, *Alpinia oxyphylla* Miq. 10 g, *Platycladus orientalis* (L.) Franco. 10 g |
| Liu et al. (2019) | Kaiqiao Xingshen Decoction (decocting pieces) | *Astragalus mongholicus* Bunge 6 g, *Angelica sinensis* (Oliv.) Diels 1.5 g, *Achyranthes bidentata* Blume 3 g, *Acorus calamus* var. angustatus Besser 3 g, *Trichosanthes kirilowii* Maxim. 5 g, *Rheum palmatum* L. 5 g, *Arisaema erubescens* (Wall.) Schott 6 g |
| Ma et al. (2018) | Pujin Keli (granule) | *Acorus calamus* var. angustatus Besser 10 g, *Salvia miltiorrhiza* Bunge 20 g, *Carthamus tinctorius* L. 10 g, |

| | | |
|---|---|---|
| | | *Curcuma longa* L. 10 g |
| Sun et al. (2017) | Xingnao Yizhi Fang (decocting pieces) | Processed *Rehmannia glutinosa* (Gaertn.) DC. 15 g, *Salvia miltiorrhiza* Bunge 15 g, *Morinda officinalis* How. 12 g, *Codonopsis pilosula* (Franch.) Nannf. 12 g, *Dioscorea oppositifolia* L. 12 g, *Pheretima aspergillum* (perrier) 6 g, *Polygala tenuifolia* Willd. 12 g, *Acorus calamus* var. angustatus Besser 9 g, *Epimedium brevicornum* Maxim. 9 g, *Cuscuta chinensis* Lam. 12 g, *Pueraria lobata* (Willd.) Ohwi 12 g, *Glycyrrhiza uralensis* Fisch. ex DC. 6 g; for excessive phlegm: add *Pinellia ternata* (Thunb.) Makino, *Trichosanthes kirilowii* Maxim. |
| Shan et al. (2017) | Nourishing Kidney and Inducing Resuscitation for Expelling Phlegm Prescription (granule) | *Acorus calamus* var. angustatus Besser 1 pack, *Polygala tenuifolia* Willd. 1 pack, *Chinemys reevesii* (Gray) 1 pack, *Cervus nippon* Temminck 1 pack, processed *Rehmannia glutinosa* (Gaertn.) DC. 1 pack, *Placenta Hominis* 1 pack, *Cuscuta chinensis* Lam. 1 pack, *Alpinia oxyphylla* Miq. 1 pack, *Cimicifuga heracleifolia* Kom. 1 pack, *Buthus martensii* Karsch 1 pack |
| Yu et al. (2016) | Huangqi Guizhi Wuwu Tang (decocting pieces) | *Astragalus mongholicus* Bunge 12 g, *Cinnamomum cassia* (L.) J. Presl 9 g, *Codonopsis pilosula* (Franch.) Nannf. 9 g, *Spatholobus suberectus* Dunn 9 g, *Alpinia oxyphylla* Miq. 9 g, *Peaonia veitchii* Lynch 6 g, *Paeonia lactiflora* Pall. 6 g, *Angelica sinensis* (Oliv.) Diels 6 g, *Poria cocos* (Schw.) Wolf 6 g, *Polygala tenuifolia* Willd. 6 g, *Whitmania acranulata* Whitman 6 g, *Pheretima aspergillum* (perrier) 6 g, *Glycyrrhiza uralensis* Fisch. ex DC. 6 g |
| Cheng et al. (2016) | High dose of *Astragalus mongholicus* (decocting pieces) | *Astragalus mongholicus* Bunge 60/90/120/150/250/300/500 g according to patient condition, *Aconitum carmichaeli* Debeaux 30 g, *Zingiber officinale* Roscoe 30 g, *Glycyrrhiza uralensis* Fisch. ex DC. 30 g, *Cornus officinalis* Siebold & Zucc. 60 g |
| Du et al. (2016) | Modified Suanzaoren (granule) | *Ziziphus jujuba* Mill. 10–15 g, *Poria cocos* (Schw.) Wolf 10–15 g, *Anemarrhena asphodeloides* Bunge 6–9 g, *Conioselinum anthriscoides* 'Chuanxiong' 6–9 g, *Cryptotympana pustulata* Fabricius 6 g, *Juncus effusus* L. 6 g, Succinum 3 g, *Glycyrrhiza uralensis* Fisch. ex DC. 3 |

| | | |
|---|---|---|
| | | g; for poor appetite: add Jiao Sanxian (*Crataegus pinnatifida* Bge. var. major N. E. Br, *Massa Fermentata* Medicinalis, *Hordeum vulgare* L.) 10–15 g; for constipation: add *Cassia obtusifolia* L. 6–9 g; for abdominal fullness: add *Citrus aurantium* L. 6–9 g, *Magnolia officinalis* Rehder & E.H. Wilson 6–9 g; for sleep bruxism: add *Phragmites communis* Trin. 15 g |
| Lou et al. (2016) | Shujinhuoluo Wan (pill) | *Acanthopanax gracilistylus* W. W. Smith, *Clematis chinensis* Osbeck, *Notopterygium incisum* Ting. ex H. T. Chang, *Siegesbeckia orientalis* L., *Arisaema erubescens* (Wall.) Schott, *Conioselinum anthriscoides* 'Chuanxiong', *Angelica biserrata* (Shan et Yuan) Yuan et Shan, *Cinnamomum cassia* (L.) J. Presl, *Chaenomeles speciosa* (Sweet) Nakai, *Angelica sinensis* (Oliv.) Diels, *Achyranthes bidentata* Blume, *Illicium difengpi* K.I.B. et K.I.M., honey (6 g per pill) |
| Lu et al. (2012) | Shenluqizhi Decoction (decocting pieces) | *Panax ginseng* C.A. Mey. 1 g, *Cervus nippon* Temminck 1 g, *Chinemys reevesii* (Gray) 1 g, Placenta Hominis 1 g, *Polygonum multiflorum* Thunb. 12 g, *Astragalus mongholicus* Bunge 20 g, *Eucommia ulmoides* Oliv. 9 g, *Cyathula officinalis* K.C. Kuan 12 g, *Achyranthes bidentata* Blume 12 g, *Pheretima aspergillum* (perrier) 9 g, *Gastrodia elata* Blume 6 g |
| Shi et al. (2015) | BuShen JianNao (capsule) | *Chinemys reevesii* (Gray), processed *Rehmannia glutinosa* (Gaertn.) DC., *Polygonum multiflorum* Thunb., *Cornus officinalis* Siebold & Zucc., *Cervus nippon* Temminck, *Panax ginseng* C.A. Mey., *Dioscorea oppositifolia* L., *Acorus calamus* var. angustatus Besser, *Polygala tenuifolia* Willd. |
| Qian et al. (2009) | Sijunzi Decoction (decocting pieces) | *Pseudostellaria heterophylla* (Miq.) Pax ex Pax et Hoffm. 15 g, *Poria cocos* (Schw.) Wolf 10 g, *Atractylodes macrocephala* Koidz. 10 g, *Glycyrrhiza uralensis* Fisch. ex DC. 6 g |
