# Supplementary figures and images for: Chinese herbal medicine for the treatment of children with cerebral palsy: a meta-analysis of randomized controlled trials with core herbs exploration
Source: Front Pharmacol. 2025 Feb 26;16:1500095. doi: 10.3389/fphar.2025.1500095 (PMC11897310; doi:10.3389/fphar.2025.1500095)

# Funnel plot

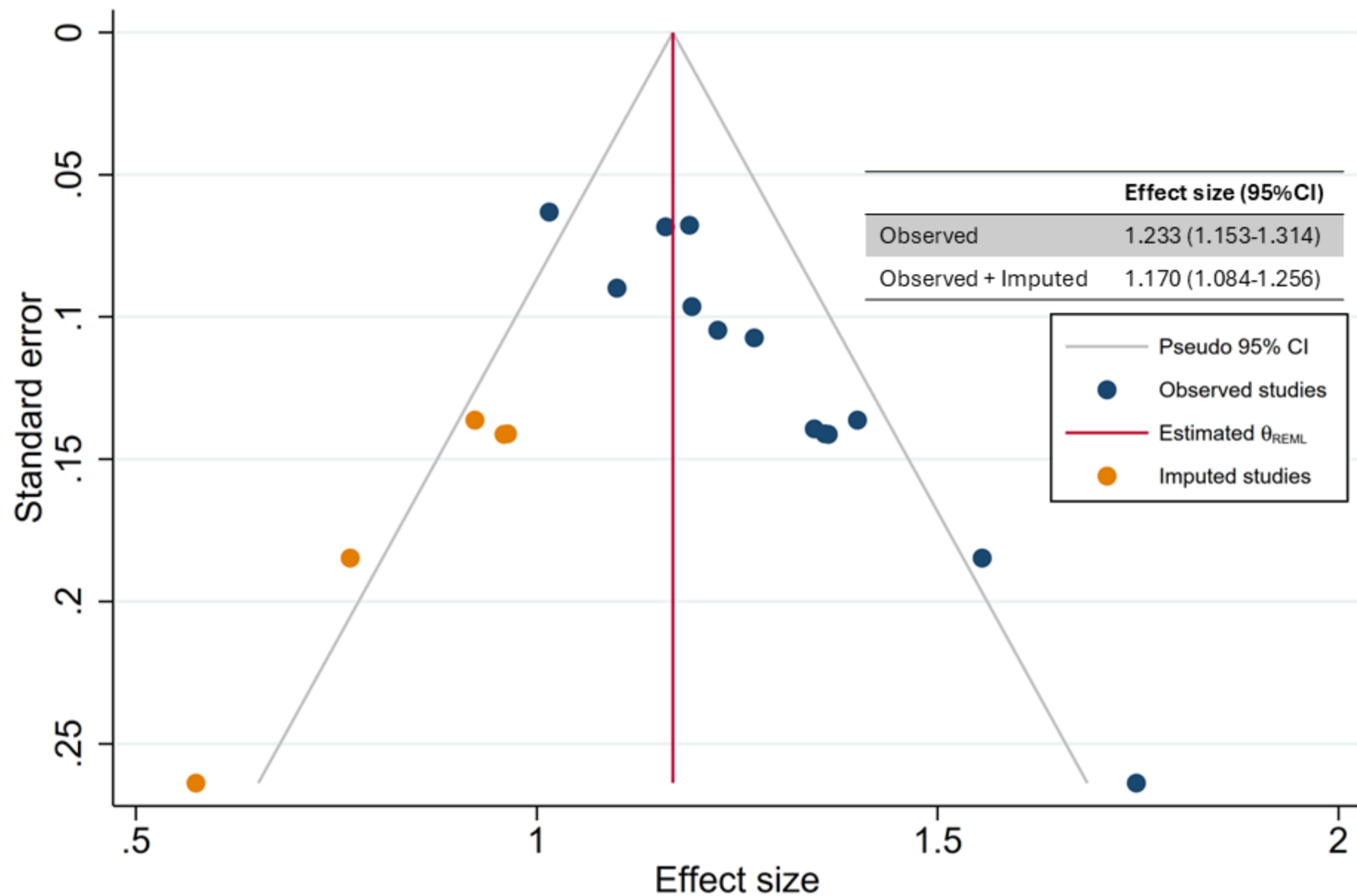

Supplement: Supplementary file 3 [file DataSheet4.pdf]

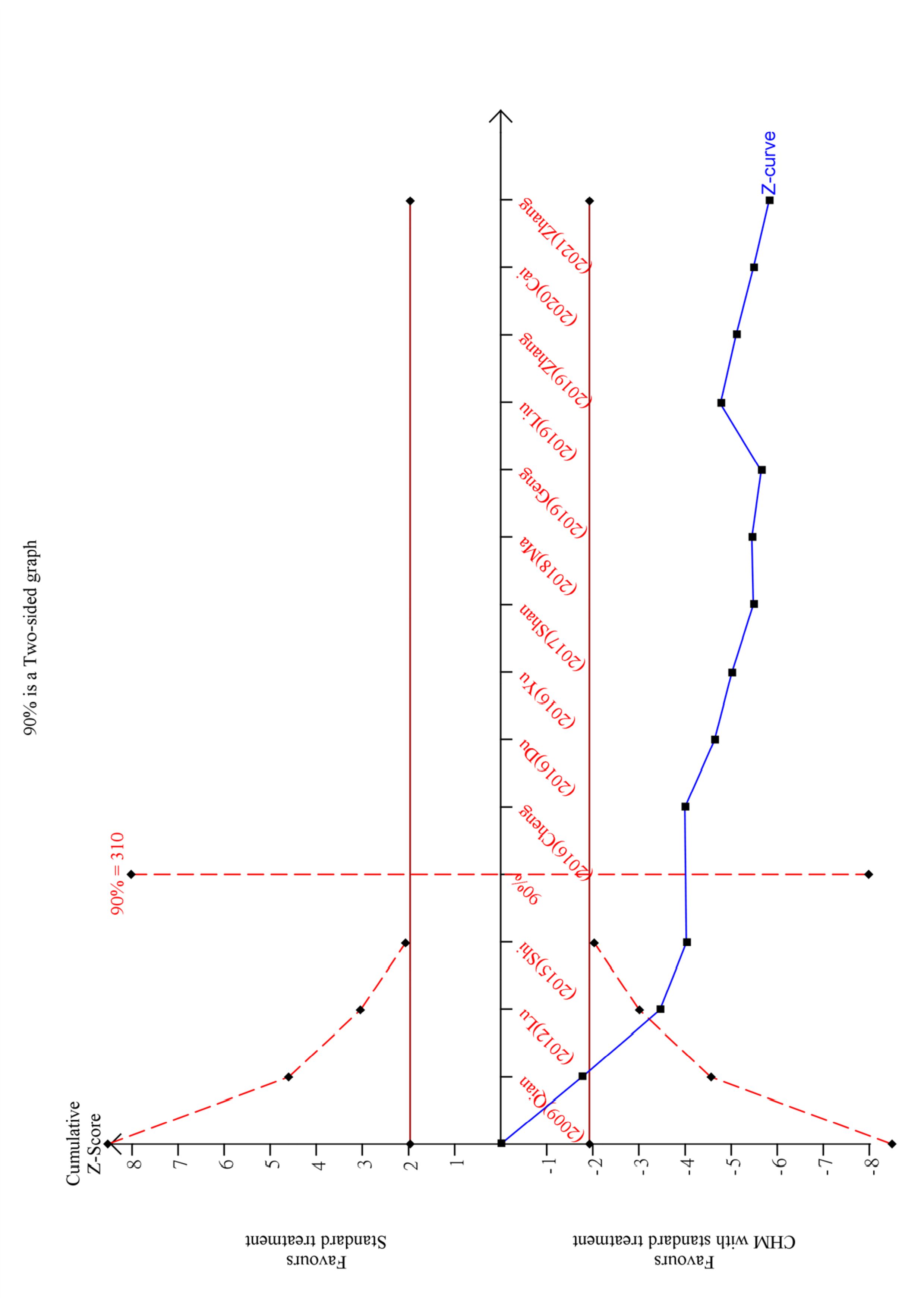

Supplement: Supplementary file 5 [file Image1.jpeg]

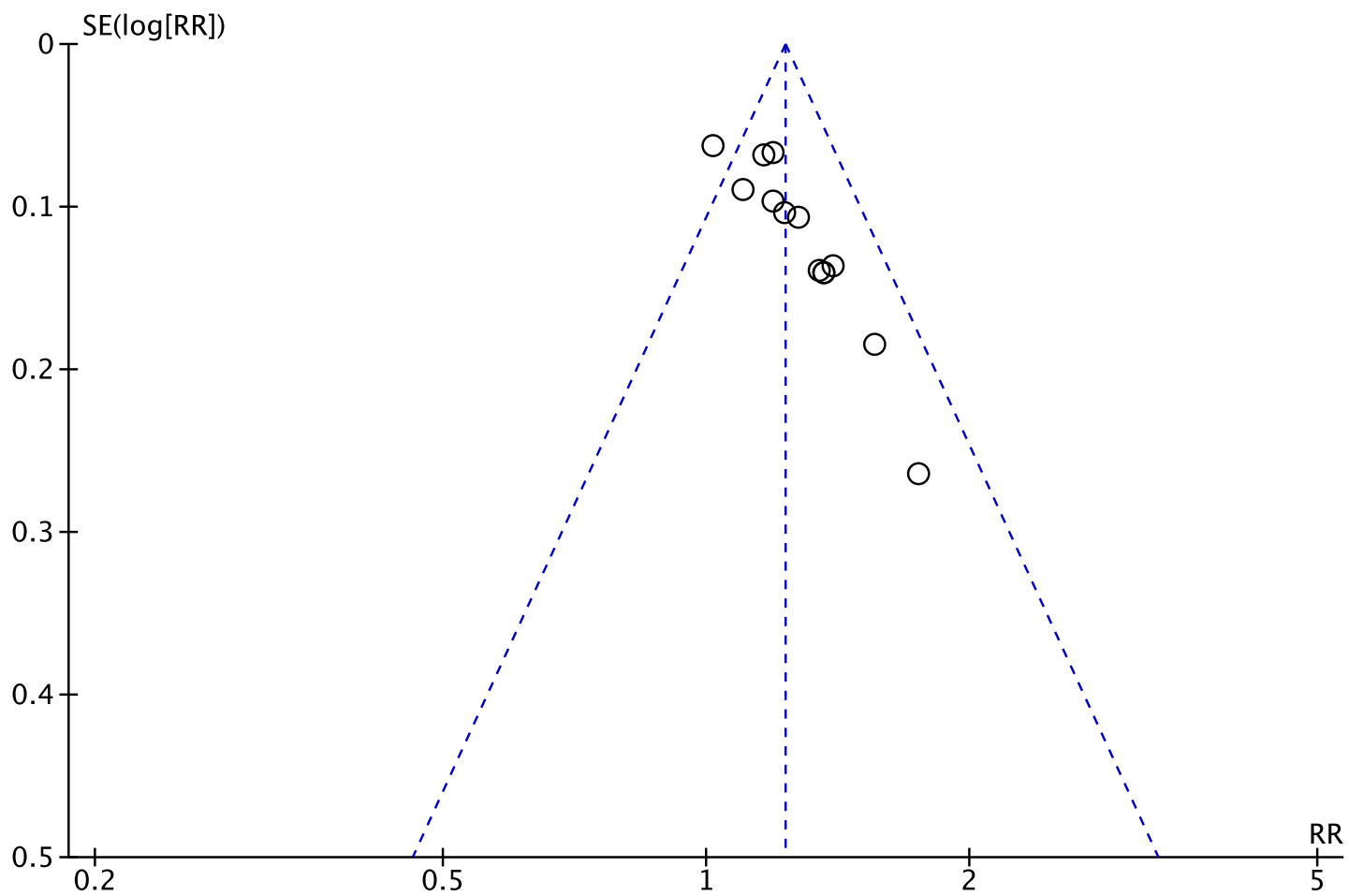

Supplement: Supplementary file 6 [file DataSheet3.pdf]
