# Supplementary material for: Chinese herbal medicine for the treatment of children with cerebral palsy: a meta-analysis of randomized controlled trials with core herbs exploration
Source: Front Pharmacol. 2025 Feb 26;16:1500095. doi: 10.3389/fphar.2025.1500095 (PMC11897310; doi:10.3389/fphar.2025.1500095)
Supplement: Supplementary file 7 [file DataSheet1.pdf]

Appendix 1. Search strategies used in the meta-analysis.

A. Search strategy used in PubMed

| | | |
|---|---|---|
| #1 | "Cerebral Palsy" [MeSH Terms] | 23,329 |
| #2 | "medicine, Chinese traditional" [MeSH Terms] OR "traditional Chinese medicine" [Text Word] OR "Herbal Medicine" [MeSH Terms] OR "medicine Korean traditional" [All Fields] OR "drugs, Chinese herbal" [MeSH Terms] OR "medicine, East Asian traditional" [MeSH Terms] OR "plants, medicinal" [MeSH Terms] OR "medicine, traditional" [MeSH Terms] OR "medicine, kampo" [MeSH Terms] OR "kampo" [Text Word] | 154,049 |
| #3 | "Randomized Controlled Trial" [All Fields] | 628,145 |
| #4 | "animals" [MeSH Terms:noexp] OR "animal" [All Fields] | 7,471,096 |
| #5 | #1 and #2 and #3 not #4 | 12 |

B. Search strategy used in Cochrane

| | | |
|---|---|---|
| #1 | MeSH descriptor: [Cerebral Palsy] explode all trees | 2,124 |
| #2 | MeSH descriptor: [Medicine, Chinese Traditional] explode all trees | 1,549 |
| #3 | MeSH descriptor: [Herbal Medicine] in all MeSH products | 243 |
| #4 | MeSH descriptor: [Medicine, Korean Traditional] explode all trees | 41 |
| #5 | Mesh descriptor: [Medicine, Kampo] explode all trees | 58 |
| #6 | #2 or #3 or #4 or #5 | 1,882 |
| #7 | MeSH descriptor: [Randomized Controlled Trail] explode all trees | 25,732 |
| #8 | Animal | 26,905 |
| #9 | #1 and #6 and #7 Not #8 | 0 |

C. Search strategy used in Embase

| | | |
|---|---|---|
| #1 | 'cerebral palsy'/exp OR 'cerebral palsy' | 52,676 |
| #2 | 'Chinese medicine'/exp OR 'herbal medicine'/exp OR 'Korean medicine'/exp OR 'kampo'/exp | 97,330 |
| #3 | 'randomized controlled trail'/exp | 770,065 |
| #4 | animal | 6,970,566 |
| #5 | #1 AND #2 AND #3 NOT #4 | 8 |

D. Search strategy used in Scopus

| | | |
|---|---|---|
| #1 | TITLE-ABS-KEY("cerebral palsy") | 47,375 |

| | | |
|---|---|---|
| #2 | TITLE-ABS-KEY(“traditional Chinese medicine” OR “herbal medicine” OR “Korean medicine” OR “kampo medicine”) | 94,651 |
| #3 | TITLE-ABS-KEY(“randomized controlled trial” OR “RCT”) | 20,246 |
| #4 | TITLE-ABS-KEY(“animal”) | 8,197,170 |
| #5 | #1 AND #2 AND #3 AND NOT #4 | 0 |

E. Search strategy used in CNKI

| | | |
|---|---|---|
| #1 | 脑瘫 + 小儿脑瘫 + 脑性瘫痪 + 脑性麻痹 + 痉挛性脑瘫 + 儿童脑瘫 + 小儿脑性瘫痪 | 61,463 |
| #2 | 中药 + 本草 + 汤 + 丸 + 散 + 方 + 颗粒 | 4,025,574 |
| #3 | 临床实验 + 临床试验+ 随机对照 + 随机对照治疗 + 随机双盲对照 + 对照实验 + 对照试验 + 临床观察 + 疗效观察 + 临床研究 | 2,921,559 |
| #4 | 针刺 | 143,757 |
| #5 | 熏蒸 | 39,326 |
| #6 | 蜡疗 | 846 |
| #7 | 药浴 | 3,714 |
| #8 | 研究进展 | 884,969 |
| #9 | #1 AND #2 AND #3 NOT #4 NOT #5 NOT #6 NOT #7 NOT #8 | 139 |

*Date of search: 31 Dec 2022

CNKI, China National Knowledge Infrastructure; RCT, randomized controlled trial.
